# Euclidean Distance Analysis Enables Nucleotide Skew Analysis in Viral Genomes

**DOI:** 10.1155/2018/6490647

**Published:** 2018-10-30

**Authors:** Formijn van Hemert, Maarten Jebbink, Andries van der Ark, Frits Scholer, Ben Berkhout

**Affiliations:** ^1^Laboratory of Experimental Virology, Medical Microbiology, Amsterdam UMC, University of Amsterdam, Amsterdam, Netherlands; ^2^Research Institute of Child Development and Education, University of Amsterdam, Amsterdam, Netherlands; ^3^Medical Microbiology, Amsterdam UMC, University of Amsterdam, Meibergdreef 9, 1105 AZ Amsterdam, Netherlands

## Abstract

Nucleotide skew analysis is a versatile method to study the nucleotide composition of RNA/DNA molecules, in particular to reveal characteristic sequence signatures. For instance, skew analysis of the nucleotide bias of several viral RNA genomes indicated that it is enriched in the unpaired, single-stranded genome regions, thus creating an even more striking virus-specific signature. The comparison of skew graphs for many virus isolates or families is difficult, time-consuming, and nonquantitative. Here, we present a procedure for a more simple identification of similarities and dissimilarities between nucleotide skew data of coronavirus, flavivirus, picornavirus, and HIV-1 RNA genomes. Window and step sizes were normalized to correct for differences in length of the viral genome. Cumulative skew data are converted into pairwise Euclidean distance matrices, which can be presented as neighbor-joining trees. We present skew value trees for the four virus families and show that closely related viruses are placed in small clusters. Importantly, the skew value trees are similar to the trees constructed by a “classical” model of evolutionary nucleotide substitution. Thus, we conclude that the simple calculation of Euclidean distances between nucleotide skew data allows an easy and quantitative comparison of characteristic sequence signatures of virus genomes. These results indicate that the Euclidean distance analysis of nucleotide skew data forms a nice addition to the virology toolbox.

## 1. Introduction

Nucleotide skew analysis [1] provides a powerful tool to visualize compositional aspects of a DNA/RNA sequence. For instance, the minimum and maximum of a G vs C skew can be used to predict the origin of replication and the location of the terminus, respectively, in prokaryotic genomes [2–4]. The GenSkew algorithm [1] calculates ratio values of the six nucleotide combinations (C vs G, G vs A, U vs G, U vs A, C vs A, and U vs C) in predefined windows and steps along a sequence. Ratio values are calculated according to (N1-N2)/(N1+N2), and hence, a positive value indicates that N1 wins over N2. Skew graphs are generally created by plotting the subsequent windows as numbers on the *X*-axis against the corresponding cumulative skew values on the *Y*-axis. In this way, we demonstrated for a representative collection of RNA viruses that the skew plots can be interpreted as “nucleotide compositional signatures” of the viral genomes and that these characteristic signatures are more prominently observed in the single-stranded regions than that in the base-paired, double-stranded regions of a viral RNA genome [5]. Likewise, we demonstrated that purine enrichment in the Zika virus RNA genome [6] is a general property of most but not all Flaviviridae and, surprisingly, prominently observed at the first position of the codons and not the silent 3^rd^ codon position (unpublished results). It is, however, difficult, time-consuming, and nonquantitative to compare different skew graphs with respect to similarities and dissimilarities. We therefore developed a simple mathematical addition to GenSkew analysis that converts skew data into a pairwise Euclidean distance matrix, which can be formatted by means of clustering into a neighbour-joining tree, facilitating the identification of putative relationships, e.g., between viral sequences. The key result of this study is that this Euclidean algorithm offers an easy and quantitative interpretation of nucleotide skew data of virus genomes. The construction of Euclidean distance trees based on skewed nucleotide compositions does not require a prior alignment of the sequences. In contrast, “classical” phylogenetic trees are modelled after accurate nucleotide alignment of the sequences. As an additional result, we demonstrate that skew distance trees and phylogenetic trees are surprisingly similar but not identical.

## 2. Materials and Methods

Nucleotide sequences of the single-stranded (ss) RNA genomes of coronavirus, picornavirus, and HIV (reference strains and unclassified species) were downloaded from the ViralZone database (http://viralzone.expasy.org/) [7, 8]. Flaviviridae were selected and classified according to [9, 10] (Berkhout and van Hemert, unpublished results). GenBank IDs are provided in the figures. Nucleotide skew analysis was done by means of the GenSkew algorithm [1], of which Dr. T. Rattei (Technische Universität München) kindly provided a version that is not restricted by the length of a sequence. For normalization purposes, the overlapping window size was set 1% of the sequence length with a step size of 20% of the window size. The skew between two nucleotides N1 and N2 is defined by the ratio (N1 − N2)/(N1 + N2), and hence, a positive value of this ratio indicates that N1 proportionally exceeds N2. If the N1 versus N2 comparison results in a negative skew value, the same but positive skew value is true for these nucleotides in the reverse order (N2 versus N1). Algorithms converting skew data into a pairwise Euclidean distance matrix are provided as Additional File S1. The multiple sequence alignment of coronaviral, flaviviral, picornaviral, and HIV genomes was obtained by means of MAFFT [11]. Other alignments and Neighbor Joining (NJ) skew distance trees were built in MEGA v7 [12]. Phylogenetic histories were inferred by using the maximum-likelihood method based on the general time reversible model of nucleotide substitution in the viral genomes [12]. A discrete gamma distribution (5 categories) was used to model evolutionary rate differences among sites. The tree with the highest log likelihood is shown. Randomization of the rubella virus RNA genome (10 consecutive cycles to ensure complete nucleotide randomization) was performed by means of the BioWeb server (http://www.cellbiol.com/scripts/randomizer/dna_protein_sequence_randomizer.php). All calculations were performed in Excel.

## 3. Results

### 3.1. Nucleotide Skew Analysis of a Single Sequence

**Figure 1:**
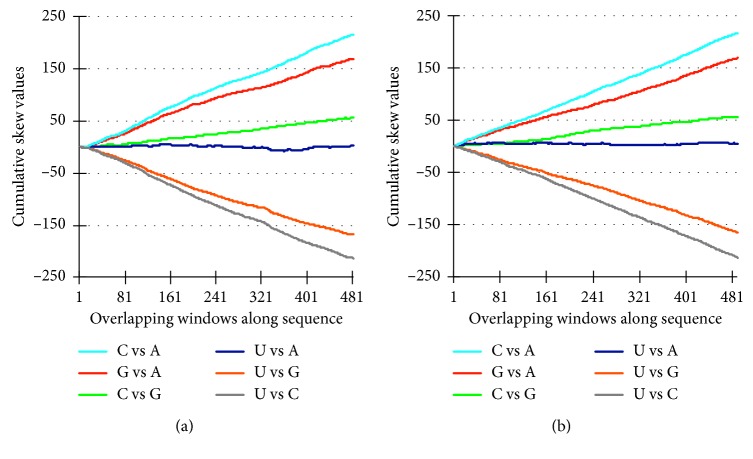
Nucleotide skew profiles of the rubella virus genome (JN635259). (a) Rubella virus. (b) 10x randomized.

We used the rubella virus with its G- and C-rich RNA genome (JN635295) to illustrate the skew plot analysis (Figure 1). Skew profiles are shown of the nucleotide sequence before Figure 1(a) and after 10 consecutive cycles of randomization Figure 1(b). The profiles are nearly identical because skew profiles are determined solely by nucleotide composition and not by the nucleotide sequence. The rubella virus RNA genome size is 9761 nucleotides, and hence, the window size and step size are set to 98 (1% of sequence length) and 20 (20% of window size), respectively, generating 489 overlapping windows from the 5′- to the 3′-end (*X*-axis) with the corresponding skew values cumulatively plotted on the *Y*-axis. The skew lines start by definition at the origin of the plot. It should be noted that, in skew language, CG does not represent a CG base pair but a comparison of the C with the G nucleotide proportions. We adopted the notation C versus G (C vs G) in the text and the figures. The nucleotides C and G win over A (steep positive slope), and the U-nucleotide loses from C and G (steep negative slope) by approximately the same proportion. C is slightly more prominent than G, and the proportions of U and A are close to equality. Importantly, the skew profiles are straight lines, which indicates that the virus-specific nucleotide bias is quite constant along the sequence of the RNA genome. Therefore, skew values at the ultimate 3′ end of the genome (position 489) represent a reliable measure of the pairwise nucleotide compositional bias of the rubella virus genome. For instance, the skew endpoint values of C vs A (216.24) and U vs C (−213.37) are very similar but with the opposite sign. The same is true for G vs A (169.79) and U vs G (−168.02), and the skew endpoint values for C vs G and U vs A are 56.39 and 2.31, respectively. For the rubella virus genome, these values can be considered a characteristic signature of the nucleotide composition and presented as a vector in a six-dimensional Euclidean space (C vs G, G vs A, U vs G, U vs A, C vs A, U vs C) = (56.39, 169.79, −168.02, 2.31, 216.24, −213.37).

The skew lines (N1 − N2)/(N1 + N2) are shown of the rubella virus genome sequence (Figure 1(a)) and the same sequence after 10 consecutive cycles of nucleotide randomization (Figure 1(b)). Overlapping windows are plotted on the *X*-axis against the corresponding skew values cumulatively on the *Y*-axis.

### 3.2. Nucleotide Skew Analysis of Unrelated Sequences

The algorithm outlined above can be applied to sets of sequences provided that one carefully preserves the order of nucleotide comparisons as well as the normalization of window and step sizes to gain ±500 cumulative skew values. The resulting vectors can be formatted into a pairwise Euclidean distance matrix and visualized by means of cluster analysis as a neighbor-joining tree. It should be emphasized that the branch nodes of the skew value trees predict equality of the nucleotide skew values among the viral species involved. In contrast, branch nodes of evolutionary trees derived by means of nucleotide substitution models mark the expectation of sequence equality—the common ancestor—of the taxa involved. Hence, only partial similarity between the two tree building methods can be expected. Here, we apply the procedure to collections of arbitrarily chosen genomes of picornaviruses, flaviviruses, coronaviruses, and HIV-1 subtypes. First, we investigated whether the Euclidean skew algorithm is able to discern differences among the four families of viral genomes. Next, the four viral families are individually subjected to the Euclidean skew analysis.

**Figure 2:**
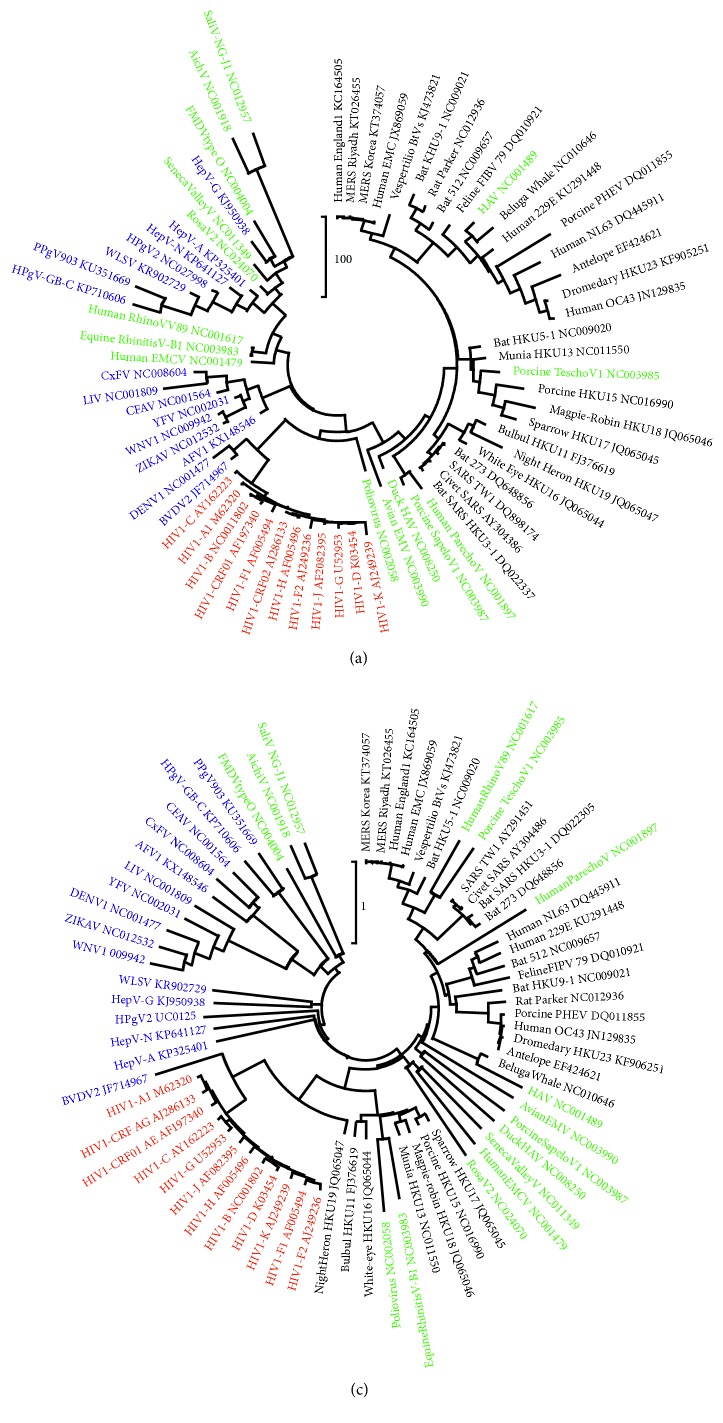
Trees of virus genomes based on nucleotide skew values (a) or nucleotide substitution (b).

Indeed, the neighbor-joining tree (Figure 2(a)) shows a consistent clustering of the HIV-1 subtypes (red), the coronaviruses (black), and the flaviviral species (blue). The picornaviruses (green) are significantly less resolved. Notably, HIV-1 genomes share A enrichment as the common feature of their nucleotide composition [13], coronavirus genomes typically tend to accumulate the U-nucleotide [14], and the Flaviviridae displays enhanced purine proportions in the first codon positions (unpublished results). The genomes of picornavirus species do not display such a prominent compositional feature. Apparently, the skew algorithm is able to discern viral families by giving credit to unique compositional features that are typical of a virus family. Viral genomes with a nucleotide composition that is more relaxed like picornavirus RNA are resolved poorly by the skew algorithm. A “classically” constructed phylogeny of the viruses shows a similar grouping (Figure 2(b)). HIV (red), coronavirus (black), and flavivirus species (blue) are clustered in separated clades, whereas representatives of picornavirus are found scattered among the flavi- and coronaviruses. With respect to salivirus, aichivirus, foot-and-mouth disease virus, and hepatitis A virus, both trees show similar topological positions, but in general, this is not the case. In addition, construction of “classical” phylogenetic trees relies a great deal on the accurate alignment of the sequences involved, which becomes less reliable as the evolutionary distance of the taxa increases. For that reason, we separately analyzed the four virus families.

Virus genomes are mentioned by short names and GenBank IDs and are colored black (coronaviruses), blue (flaviviruses), green (picornaviruses), and red (HIVs). The trees were constructed by means of neighbor-joining clustering (NJ, Figure 2(a)) and by maximum likelihood phylogeny (ML, Figure 2(b)). The scale bars are in skew units (Figure 2(a)) or estimate the number of nucleotide substitutions per site (Figure 2(b)).

### 3.3. Nucleotide Skew Analysis of Related Sequences

#### 3.3.1. Coronavirus

**Figure 3:**
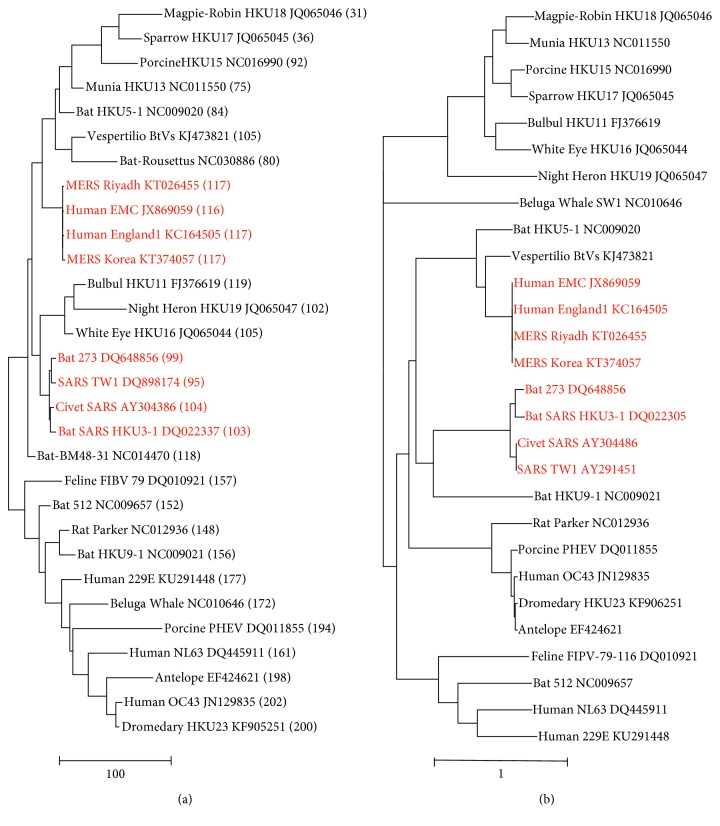
Trees of coronavirus genomes based on nucleotide skew values (a) or nucleotide substitution (b).

Coronavirus RNA genomes show high U and low C proportions that are quite variable and in fact act like communicating vessels [14]. Indeed, a bipartition can be observed in the cluster tree based on Euclidean skew distances (Figure 3(a)). The upper group containing the MERS and SARS (in red) isolates is characterized by relatively low U vs C skew values varying from 120 to 31. The lower group with the human 229E, NL63, and OC43 coronaviruses displays U vs C skew values increasing from 140 to 201. Apparently, the opposite movement of the U and C proportions in coronaviral genomes is a distinctive feature, and hence, an important parameter in shaping coronaviral phylogenies is based on nucleotide substitution (Figure 3(b)). We included two unclassified virus isolates—marked in italic—to investigate whether the method can contribute to an initial virus classification. The unclassified *Bat-Rousettus* isolate is found near the *Vespertilio* coronavirus, and the unclassified *Bat-BM48-31* isolate is at the root position of in the upper clade.

Coronavirus genomes are mentioned by short names and GenBank IDs. Clustering of MERS and SARS coronaviruses is indicated by coloring in red. Numbers in brackets indicate U versus C skew values. Two clades differing by U vs C skew values can be discerned in the skew-based tree, the upper clade with U vs C proportions decreasing from 120 to 31 and the lower clade with U vs C proportions decreasing from 201 to 140. The two unclassified isolates are italicized. The trees were constructed by means of neighbor-joining clustering (NJ, Figure 3(a)) and by maximum likelihood phylogeny (ML, Figure 3(b)). The scale bars are in skew units Figure 3(a) or estimate the number of nucleotide substitutions per site Figure 3(b).

#### 3.3.2. Flavivirus

**Figure 4:**
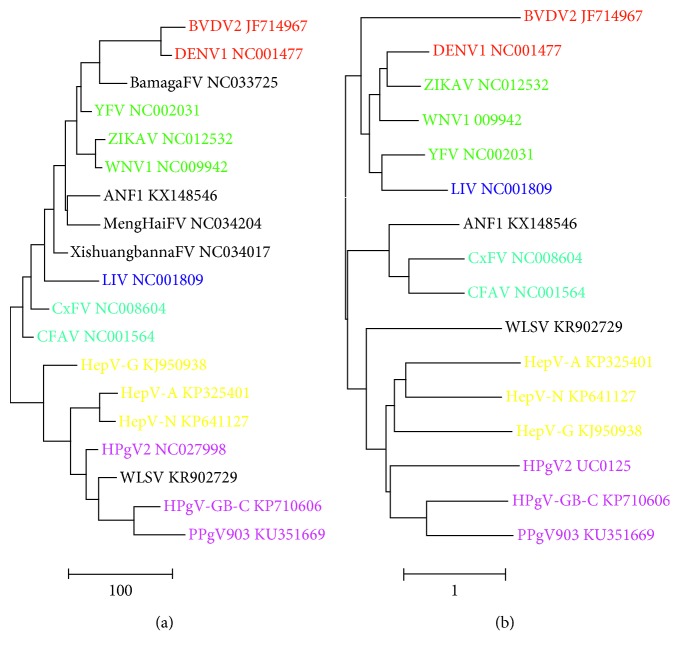
Trees of flavivirus genomes based on nucleotide skew values (a) or nucleotide substitution (b).

The Flaviviridae represent an extensive group of ssRNA viruses with a diverse nucleotide composition. Yet, the pairwise distance tree based on skew values (Figure 4(a)) is in remarkable agreement with the topology obtained by means of evolutionary models (Figure 4(b)) [9]. Dengue virus (DENV), bovine viral diarrhea virus (BVDV), yellow fever virus (YFV), Zika virus (ZIKAV), West Nile virus (WNV) species, and the unclassified Bamaga flavivirus are clustered into the clade of mosquito-borne flaviviruses. The unclassified *Anopheles*, Menghai, and Xishuangbanna flaviviruses map in between the mosquito-borne viruses and the tick-borne representative (louping ill virus, LIV). The insect-specific culex flavivirus (CxFV) and cell fusing agent virus (CFAV) cluster together. Finally, the skew algorithm distinguishes the hepaciviruses from the pegiviruses (including Wenling shark virus, WLSV) consistent with more classical phylogeny [10].

Flavivirus genomes are mentioned by short names and GenBank IDs. Representatives of the flaviviruses are colored red (dengue virus and pestivirus), green (flavivirus), blue (tick-borne flavivirus), light-blue (insect-specific flavivirus), yellow (hepacivirus), and magenta (pegivirus). The five unclassified flavivirus genomes are italicized. The trees were constructed by means of neighbor-joining clustering (NJ, Figure 4(a)) and by maximum likelihood phylogeny (ML, Figure 4(b)). The scale bars are in skew units (Figure 4(a)) or estimate the number of nucleotide substitutions per site (Figure 4(b)).

#### 3.3.3. Picornavirus

**Figure 5:**
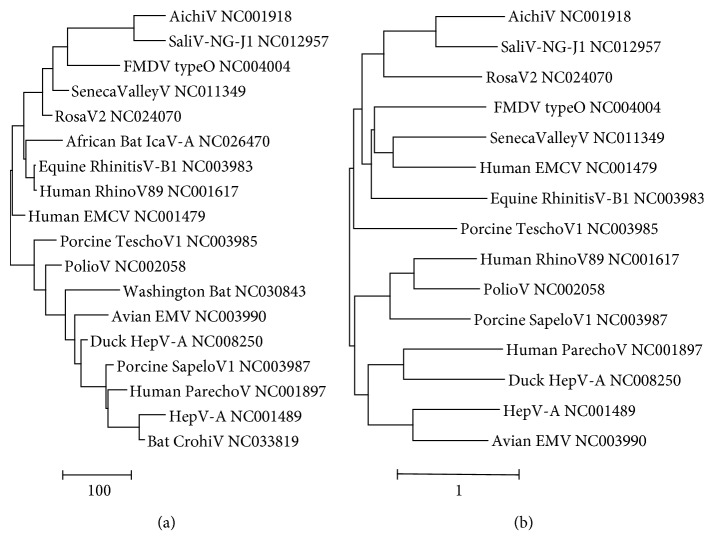
Trees of picornavirus genomes based on nucleotide skew values (a) or nucleotide substitution (b).

The skew cluster tree of picornaviruses (Figure 5(a)) also matches the tree topology based on nucleotide substitution (Figure 5(b)). As an exception, the rhinoviruses of humans and equines are close neighbors based on skew values that are separated more distantly in the standard evolutionary tree. Two of the three unclassified picornaviruses (Washington bat and bat crohivirus) map in the cluster of avian EMV and hepatitis A virus. The unclassified African bat icavirus takes a position in between rosavirus 2 and two rhinoviruses.

Picornavirus genomes are mentioned by short names and GenBank IDs. The three unclassified picornavirus genomes are italicized. The trees were constructed by means of neighbor-joining clustering (NJ, Figure 5(a)) and by maximum likelihood phylogeny (ML, Figure 5(b)). The scale bars are in skew units (Figure 5(a)) or estimate the number of nucleotide substitutions per site (Figure 5(b)).

#### 3.3.4. HIV

**Figure 6:**
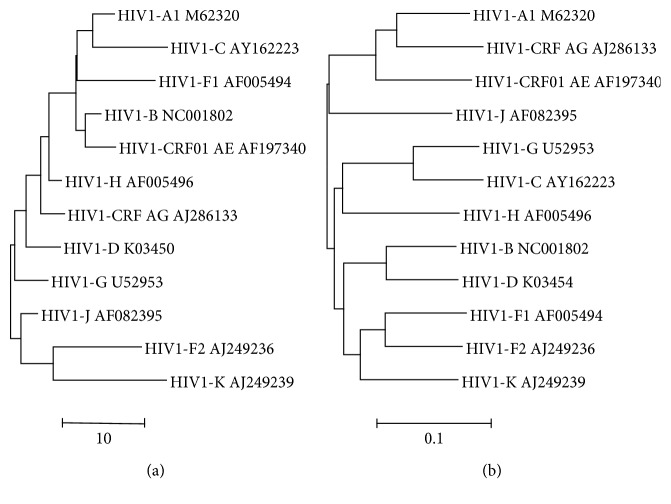
Trees of HIV-1 subtype genomes based on nucleotide skew values (a) or nucleotide substitution (b).

The reference sequences of HIV-1 subtypes show much less compositional divergence than corona-, flavi-, and picornaviruses as indicated by the approximately 10x smaller size of the scale bar (Figure 6). In fact, the nucleotide skew profiles are nearly identical to the average skew value with small standard deviation (Table 1). The well-known A-accumulation in HIV-1 genomes [13] is prominently demonstrated by the negative values for G vs A, U vs A, and C vs A (Table 1: AVG: −101.30, −118.86, and −171.62, respectively). A-accumulation occurred mainly at the expense of C (C vs G: 77.15; U vs C: 59.48). Similar calculations for average skew values and standard deviations of coronavirus, flavivirus, and picornavirus genomes did not provide useful AVG and StDev data (Table 1).

HIV-1 subtype genomes are mentioned by subtype and GenBank IDs. The trees were constructed by means of neighbor-joining clustering (NJ, Figure 6(a)) and by maximum likelihood phylogeny (ML, Figure 6(b)). The scale bars are in skew units (Figure 6(a)) or estimate the number of nucleotide substitutions per site (Figure 6(b)).

Differences can be observed between the Euclidean skew tree of HIV-1 subtypes and the HIV-1 topology based on regular nucleotide substitution models (Figure 6). For instance, the evolutionary tree indicates a close relationship of F1 and F2 strains and between both HIV-1-CRF isolates, which is not observed in the skew-based tree. The dominant A-pressure in HIV may drive the compositional signatures of different HIV sequences towards similar skew values, by which the resolving power of the Euclidean skew algorithm becomes insufficient for a proper discrimination among HIV-1 subtypes. In addition, different sequences may have similar skew profiles. For example, randomization of a sequence does not alter the skew profile of the nucleotides (Figure 1). Finally, unclassified HIV-1 subtypes were not found in the ViralZone database.

## 4. Discussion

Pairwise Euclidean distance analysis facilitates an easy initial interpretation of the relationships among viral sequences based on skew values as a nucleotide compositional signature. The algorithm distinguishes related and unrelated sequences, particularly in case of compositional features that are characteristic for a certain virus family (e.g., A-accumulation in HIV, the U-C balance in coronavirus genomes, and the purine enrichment at the first codon position in flaviviral genomes). Within a family of viral genomes, the topology based on Euclidean skew distances is surprisingly similar (but not identical) to the topology based on regular evolutionary analysis (coronavirus, flavivirus, and picornavirus). This is remarkable because branch nodes in skew-based trees predict equal skew values of both sequences, and, in contrast, branch nodes in evolutionary trees mark nucleotide sequence equality of the two taxa. The resolving power of skew value analysis is diminished at high sequence similarity as shown for HIV-1 subtypes. This is due to the prominent A-pressure in all HIV genomes [15].

The key focus of this research was the Euclidean analysis of nucleotide skew data among virus genomes. In addition, we demonstrated similarity of skew data-based cluster trees with “classical” maximum-likelihood phylogenetic trees constructed via a general time reversible (GTR) model of nucleotide substitution during evolution. Confidence of ancestral nodes in “classical” trees is usually attained by means of bootstrap analysis, which requires repetitive resampling of sites in the original sequence alignment. Each bootstrap replicate generates a tree, and a consensus tree is constructed based on the collection of replicates. Each node in this consensus tree is decorated with the frequency number (%) that reflects the level of bootstrap support. It is evident that a skew-based pairwise distance matrix cannot be used for bootstrap analysis simply due to the absence of a sequence alignment. Also, resampling of sites in a sequence prior to skew analysis does not affect the skew data vector (Figure 1). Future investigation may reveal whether the building of skew-based trees may serve as a valuable addition to many sequence-independent methods of evolutionary tree reconstruction.

## 5. Conclusions

Euclidean skew analysis supports the study of nucleotide compositional features and facilitates the classification of unclassified viral sequences as nearest neighbors of classified ones. Compared with methods based on nucleotide substitution models, (Euclidean) skew analysis is very robust in that it tolerates sequence errors and shifts in reading frames.

## Figures and Tables

**Table 1:** Mean skew values of coronavirus, flavivirus, picornavirus, and HIV-1 subtype RNA genomes. HIV-1 subtype RNAs display much lower StDEV of skew values compared with those of the other viruses.

Skew values		C vs G	G vs A	U vs G	U vs A	C vs A	U vs C
Coronavirus	AVG	−29.09	−58.05	101.36	40.51	−86.34	124.36
	StDev	46.67	33.47	21.40	24.00	43.15	45.22
Flavivirus	AVG	−43.38	38.95	−44.53	−5.01	−3.66	−1.19
	StDev	29.92	53.33	32.36	59.98	71.05	25.50
Picornavirus	AVG	9.48	−32.05	37.12	2.81	−24.10	26.49
	StDeV	62.05	45.85	40.06	39.51	95.34	81.95
HIV	AVG	−77.15	−101.30	−17.95	−118.86	−171.62	59.48
	StDEV	3.82	7.00	3.46	6.20	7.32	5.26

## Data Availability

The relevant databases ViralZone and GenBank used to support the findings of this study are included within the article (Materials and Methods). The GenBank IDs of the sequences are provided in the figures. The software used is mentioned by the references as provided by MAFFT and MEGA. Nucleotide randomization was performed by a BioWeb server application. The GenSkew algorithm was provided by Dr. T. Rattei. A web server version can be found at http://genskew.csb.univie.ac.at/.

## Supplementary Materials

Supplementary Materials Supplementary File S1: Macro in Excel and in R code converting skew values into a pairwise distance matrix.
