# Supplementary material for: Euclidean Distance Analysis Enables Nucleotide Skew Analysis in Viral Genomes
Source: Comput Math Methods Med. 2018 Oct 30;2018:6490647. doi: 10.1155/2018/6490647 (PMC6232797; doi:10.1155/2018/6490647)
Supplement: Supplementary Materials — Supplementary File S1: Macro in Excel and in R code converting skew values into a pairwise distance matrix. [file 6490647.f1.docx]

**Additional File S1:**

Macro in Excel and in R code converting skew values into a pairwise distance matrix

Public Sub dist_matrix()

Dim n As Integer, V As Range, m As Range

Dim i As Integer, j As Integer

ActiveSheet.Cells(1, 1).Select

n = Cells(1, 1).CurrentRegion.Rows.Count

Set V = Range(Cells(2, 1), Cells(n, 1))

Set m = Range(Cells(2, 9), Cells(n, n + 7))

For i = 1 To n - 1

m.Cells(0, i) = V(i, 1)

Next i

For i = 1 To n - 1

For j = 1 To n - 1

m(i, j) = euclid(V, i, j)

Next j

Next i

End Sub

Public Function euclid(R As Range, k As Integer, l As Integer) As Single

Dim S As Single

S = 0

For m = 2 To 7

S = S + (R(k, m) - R(l, m)) * (R(k, m) - R(l, m))

Next m

euclid = Sqr(S)

End Function

----------------------------------------------------------------------

# R code to convert skew values read from into a pairwise distance matrix

# If "xlsx" package has not been installed, install "xlsx" package to read Excel files.

if(!("xlsx" %in% installed.packages()[,"Package"])) install.packages("xlsx", repos = "http://cran.xl-mirror.nl/")

library(xlsx)

# Select the Excel file containing the skew values. The first column should be the labels

dataFile <- file.choose()

skewValues <- read.xlsx(dataFile, 1, header = TRUE)

labels <- as.character(skewValues[[1]])

# Compute the pairwise distance matrix

pairwiseDistance <- as.matrix(dist(skewValues[, -1]))

dimnames(pairwiseDistance) <- list(labels, labels)

# Write the pairwise distance matrix into and Excelfile

write.xlsx(x = pairwiseDistance, file = file.choose())
